# Trojan Horse Transit Contributes to Blood-Brain Barrier Crossing of a Eukaryotic Pathogen

**DOI:** 10.1128/mBio.02183-16

**Published:** 2017-01-31

**Authors:** Felipe H. Santiago-Tirado, Michael D. Onken, John A. Cooper, Robyn S. Klein, Tamara L. Doering

**Affiliations:** a Department of Molecular Microbiology, Washington University School of Medicine, St. Louis, Missouri, USA; b Department of Biochemistry and Molecular Biophysics, Washington University School of Medicine, St. Louis, Missouri, USA; c Department of Medicine, Washington University School of Medicine, St. Louis, Missouri, USA; Johns Hopkins Bloomberg School of Public Health

## Abstract

The blood-brain barrier (BBB) protects the central nervous system (CNS) by restricting the passage of molecules and microorganisms. Despite this barrier, however, the fungal pathogen *Cryptococcus neoformans* invades the brain, causing a meningoencephalitis that is estimated to kill over 600,000 people annually. Cryptococcal infection begins in the lung, and experimental evidence suggests that host phagocytes play a role in subsequent dissemination, although this role remains ill defined. Additionally, the disparate experimental approaches that have been used to probe various potential routes of BBB transit make it impossible to assess their relative contributions, confounding any integrated understanding of cryptococcal brain entry. Here we used an *in vitro* model BBB to show that a “Trojan horse” mechanism contributes significantly to fungal barrier crossing and that host factors regulate this process independently of free fungal transit. We also, for the first time, directly imaged *C. neoformans*-containing phagocytes crossing the BBB, showing that they do so via transendothelial pores. Finally, we found that Trojan horse crossing enables CNS entry of fungal mutants that cannot otherwise traverse the BBB, and we demonstrate additional intercellular interactions that may contribute to brain entry. Our work elucidates the mechanism of cryptococcal brain invasion and offers approaches to study other neuropathogens.

## IMPORTANCE

The fungal pathogen *Cryptococcus neoformans* invades the brain, causing a meningoencephalitis that kills hundreds of thousands of people each year. One route that has been proposed for this brain entry is a Trojan horse mechanism, whereby the fungus crosses the blood-brain barrier (BBB) as a passenger inside host phagocytes. Although indirect experimental evidence supports this intriguing mechanism, it has never been directly visualized. Here we directly image Trojan horse transit and show that it is regulated independently of free fungal entry, contributes to cryptococcal BBB crossing, and allows mutant fungi that cannot enter alone to invade the brain.

## INTRODUCTION

Fungal infections of the central nervous system (CNS) cause 1.5 million deaths every year worldwide (1). The major cause is the basidiomycete *Cryptococcus neoformans* (2), a ubiquitous environmental yeast (3, 4). Inhalation of this pathogen leads to pneumonia, which in healthy people either is resolved or remains asymptomatic. In the setting of immunocompromise, however, *C. neoformans* disseminates, with specific tropism for the CNS. To enter the brain, *C. neoformans* must cross the blood-brain barrier (BBB), which protects the CNS from chemical and infectious damage (5). Potential routes of cryptococcal entry include (i) direct fungal interactions with brain endothelial cells, leading to endocytosis and subsequent transcytosis of free fungi (6–9); (ii) disruption of BBB endothelial cell junctions, allowing paracellular passage of free fungi (10–13); and (iii) “Trojan horse” crossing, where fungi traverse the BBB within infected phagocytes (14–17), either transcellularly or paracellularly. However, whether all of these routes are used, the mechanistic details of the routes, and their relative contributions to brain infection are not known.

Evidence that *C. neoformans* uses a Trojan horse mechanism to traverse the BBB is primarily derived from indirect studies *in vivo*, including histological observation of fungi within phagocytes in brain microvasculature and parenchyma (18) and adoptive transfer experiments using infected macrophages (14, 16). In the adoptive transfer studies, intravenous (i.v.) administration of infected macrophages increased brain burden compared to infection with free cryptococci (14, 16). These experiments strongly suggest that phagocyte association provides a dissemination advantage, which could occur either upstream of CNS entry or at the point of BBB traversal. However, interpretation of these studies is complicated by the long intervals between infection and brain harvest, particularly because cryptococci grow both inside and outside host cells and can move between the two compartments while maintaining viability. Interpretation was also complicated in adoptive transfer studies where a heterologous population of macrophages, including free, adherent, and internalized cryptococci, was introduced into mice; *in vivo*, there is no way to track which population of fungi leads to the observed brain burden, so the route of BBB crossing cannot be determined.

Recently, the Trojan horse pathway was examined *in vitro* using a mixture of fungi and monocytes (including free, adherent, and internalized fungi) and a Transwell BBB model. At one day after addition of this mixture, monocytes containing fungi were observed in the lower chamber, suggesting that Trojan horse crossing had occurred (17). As with the earlier *in vivo* experiments, however, the mixed population and the potential for a long incubation to allow multiple cellular interactions (e.g., exit from phagocytes, free fungal crossing, and reuptake in the lower chamber) make it difficult to reach a definitive conclusion about the occurrence of Trojan horse transit in these studies.

Here we combined flow cytometry, fluorescent and live-cell microscopy, and several BBB models to directly demonstrate Trojan horse crossing. We further showed that it occurs via transcellular pore formation, is regulated independently of free fungal entry, and allows mutant fungi that cannot cross alone to invade the brain. Our results conclusively demonstrate the ability of *C. neoformans* to exploit human phagocytes as Trojan horses, contribute significantly to our knowledge of cryptococcal brain entry, and break new ground in the area of BBB transmigration of pathogens.

## RESULTS

### Isolation of macrophages loaded with single fungi.

**FIG 1:**
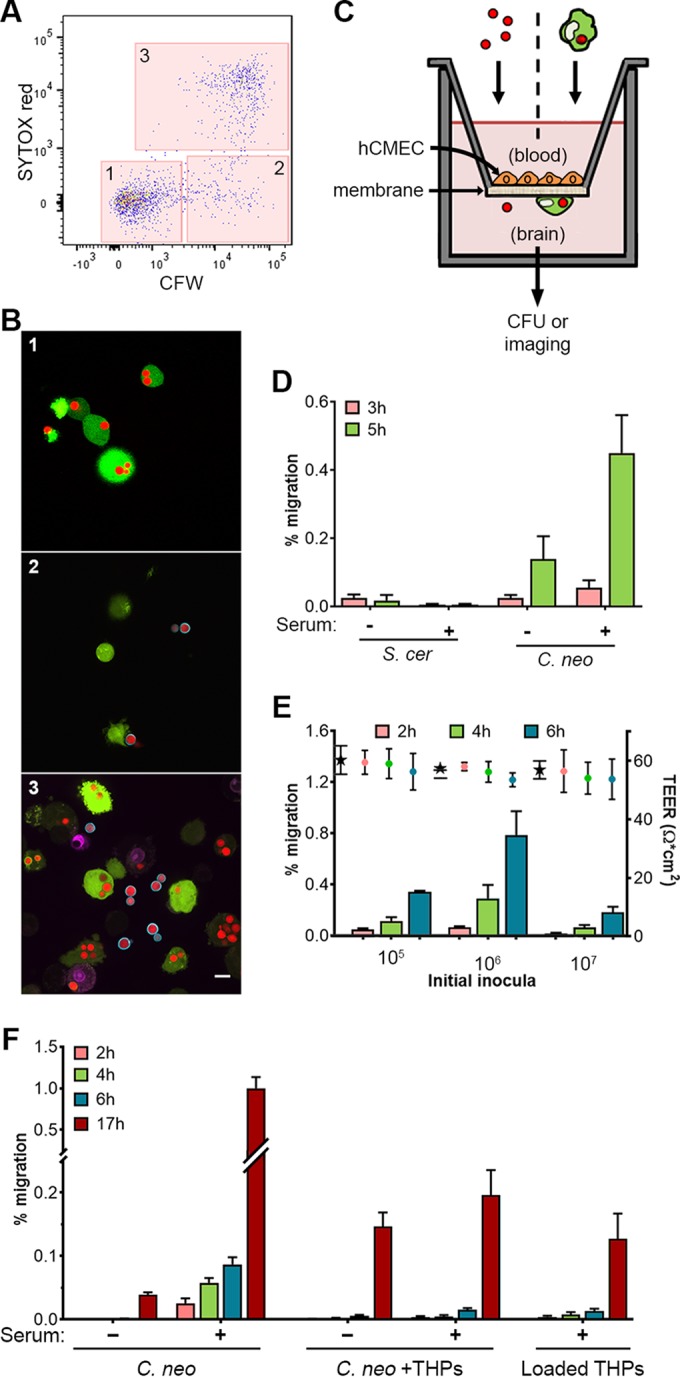
Isolation of loaded macrophages and crossing of the BBB by *C. neoformans*. (A) Final phase of the sorting strategy used to isolate phagocytes that were healthy (negative for SYTOX dye) and devoid of external fungi (negative for CFW); the complete strategy is shown in Fig. S1. The boxes corresponding to the gates are labeled as follows: gate 1, healthy phagocytes with only internal fungi; gate 2, healthy phagocytes that have external fungi (with or without internal fungi); gate 3, dead or damaged phagocytes. Results are representative of results of 69 independent analyses. (B) Cells collected from the gates shown in panel A (all shown at the same scale; bar = 10 µm). Images are representative of results of 4 independent sorting studies where cells were examined microscopically. (C) Diagram of the BBB model used in this study. (D) Transit of free *S. cerevisiae* (*S. cer*) or *C. neoformans* (*C. neo*) in the absence or presence of serum; means and standard deviations (SD) of results are shown for one of two similar studies. (E) Mean and SD values over time for transit (bars, left axis) and TEER (points, right axis), for various starting inocula of opsonized *C. neoformans*. Stars represent TEER values at 0 h. Results from one of two similar studies are shown. (F) Time-dependent transmigration of free fungi, a 1:1 mix of free fungi and empty THPs, and fungus-loaded THPs (1 to 1.49 fungi/host cell). Means and standard errors of the means (SEM) are shown for results of one of seven similar independent experiments. Values plotted for all time course transit assays are cumulative values.

To rigorously compare the BBB passage of free fungi to that of internalized fungi, we modified a flow cytometry strategy for the isolation of *C. neoformans*-infected phagocytes (19), using *C. neoformans* strain KN99α and cells of the human monocytic cell line THP-1 (THP). After incubating mCherry-expressing KN99α (see Materials and Methods) with DFFDA-stained THPs, we applied a sorting strategy (Fig. 1A; see also Fig. S1 in the supplemental material) that additionally used SYTOX red as a host cell viability marker and calcofluor white (CFW) as an impermeant stain to identify externally associated fungi. This allowed us to routinely isolate the desired loaded macrophage population, consisting of healthy host cells, devoid of externally adherent or free fungi (Fig. 1A; box 1), with a count of one yeast per cell on average (Fig. S1A). This population is shown in Fig. 1B (panel 1) and is compared to populations of phagocytes with externally adherent fungi stained by CFW (Fig. 1A; box 2; Fig. 1B, panel 2) and phagocytes with compromised viability stained by SYTOX red (Fig. 1A; box 3; Fig. 1B, panel 3).

10.1128/mBio.02183-16.1 FIG S1  Optimization of THP-1 loading and sorting strategy for isolation of singly loaded phagocytes. (A) Fungi were incubated with THPs at various MOIs for quantitation of the number of internal fungi/host cell by confocal microscopy. (B) The same conditions were assessed to determine phagocytic and adherence indices (number of cell-associated fungi/100 host cells). (C to F) Sorting strategy, applied to optimized uptake reactions, to exclude free fungi (near origin) and dead or damaged host cells (at left) (C); to select single particles (D); to exclude unassociated host cells (left, stained only with DFFDA) and fungi (lower right, stained only with mCherry) (E); and to collect the desired population (region 1) of cells that were differentiated from CFW^+^ cells bearing adherent external fungi (region 2) and SYTOX^+^ damaged cells (region 3) (F). Download FIG S1, TIF file, 1.2 MB. Copyright © 2017 Santiago-Tirado et al. 2017 Santiago-Tirado et al. This content is distributed under the terms of the Creative Commons Attribution 4.0 International license.

### BBB transit of free fungi and loaded macrophages.

*In vitro* models of the BBB have previously been used to study transit of free cryptococci (20, 21). We generated model BBB (BBB^M^) by seeding the human cerebral microvascular endothelial cell line hCMEC/D3 (22, 23) on top of permeable inserts in transwell plates (Fig. 1C). We monitored barrier development and integrity by measuring transendothelial electrical resistance (TEER) across the monolayer; once it had stabilized (5 to 6 days), we added particles of interest to the top chamber (representing the blood side of the BBB) and monitored transmigration by assaying samples from the lower chamber (representing the brain side) for CFU. Free *C. neoformans* crossed the BBB^M^ in a time-dependent manner, consistent with earlier results (20, 21); this was enhanced by the presence of human serum, suggesting that both opsonin-dependent and opsonin-independent mechanisms contribute to this process (Fig. 1D). In contrast, *Saccharomyces cerevisiae*, despite its similar size and shape, showed minimal traversal that was not influenced by serum.

We observed the highest percentage of transmigration in our assays at a fungus/host cell ratio of ~6 (Fig. 1E; 10^6^ fungi), which is the ratio that we used for subsequent studies. Higher levels of inocula did not increase this value, suggesting the presence of a saturable receptor or other limiting host factor. Barrier integrity was maintained over time with all inocula tested, as shown by constant TEER values (Fig. 1E, right axis). This suggests a minor role for paracellular traversal due to BBB^M^ compromise; instead, free fungi probably cross primarily via a transcellular process (see below).

We next compared the levels of transmigration of free serum-opsonized cryptococci and equal numbers of cryptococci contained within phagocytes; as a control to ensure that the phagocytes alone did not perturb the barrier, we assayed the same number of free fungi mixed 1:1 with uninfected phagocytes immediately prior to assay. Under standard conditions, ~10-fold more opsonized *C. neoformans* fungi crossed free than internalized in macrophages (Fig. 1F) (*P* < 0.001). Interestingly, adding empty macrophages (“*C. neo* + THPs”) inhibited traversal of free fungi (*P* = 0.001). This may reflect competition for free fungi between endothelial cells, characterized by low rates of endocytosis (22), and professional phagocytes (THPs). During the assay, the latter might have engulfed most of the free fungi, preventing them from interacting with endothelial cells to initiate transcellular crossing and yielding a traversal rate similar to that of the loaded macrophages.

### Direct visualization of Trojan horses associated with model BBB.

**FIG 2:**
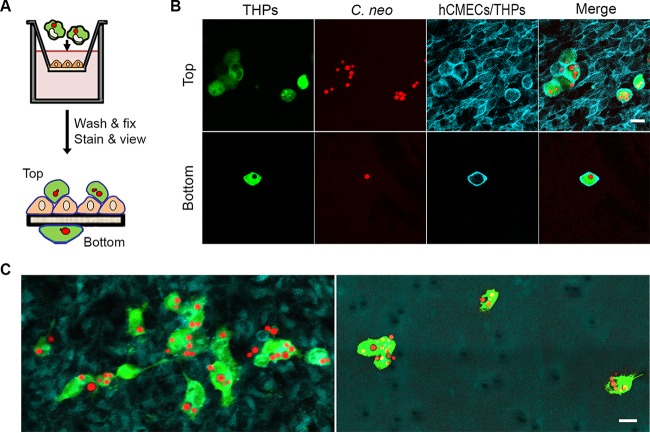
Visualization of Trojan horse crossing. (A) Experimental design for visualization of both sides of the permeable membrane. (B) A field of view showing loaded macrophages associated with the endothelial monolayer (top row) and the bottom of the porous membrane with loaded macrophages alone (bottom row). Both mammalian cell types were stained with CellMask plasma membrane dye (blue); THPs were further stained with DFFDA from the flow sorting (green) and contained mCherry-stained fungi (red). Scale bar, 10 μm. (C) Merged images from a larger field of view, again showing all cell types on the top of the membrane (left), whereas only loaded phagocytes are visible on the bottom (right). Scale bar, 20 μm. Images are representative of multiple fields from two independent experiments, each with three independent time points. The fields shown are from a 1-h time point. We observed loaded macrophages associated with the bottom of the membrane most frequently early in the incubation period (more at 1 h than at 2 to 3 h; not shown); this may represent a time-dependent loss of phagocyte viability or ability to initiate transit. See Movie S1 for another example.

Previous studies of Trojan horse crossing have been limited by the use of mixed cell populations and the lack of evidence that fungi were maintained within host phagocytes during actual barrier traversal. Our isolation of loaded macrophages devoid of externally associated cells or free fungi addressed the first concern, and we turned to direct imaging to address the second. We performed 18-h assays with loaded macrophages and imaged both sides of the cell culture inserts (Fig. 2A). Confocal microscopy of the top revealed an endothelial cell layer with abundant associated loaded macrophages (Fig. 2B, top row, and 2C, left). We also observed loaded macrophages associated with the bottom (“brain” side) of the permeable membranes (Fig. 2B, bottom row, and 2C, right). Because free *C. neoformans* fungi do not associate with the cell-free bottom surface of the membrane, the fungi in these macrophages must have crossed the barrier within them, thus directly demonstrating Trojan horse crossing. Movie S1 shows consecutive confocal sections of a field with loaded macrophages on both sides of the membrane.

10.1128/mBio.02183-16.2 MOVIE S1  A Z-stack of confocal slices 5 µm apart across the permeable membrane of a model BBB. Loaded macrophages were added to the top of an hCMEC monolayer and incubated for 1 h prior to washing and fixation performed as described in Materials and Methods. The first half of the movie shows *xyz* views, while the second half shows the same images rendered as a three-dimensional (3D) object with rotation. Download MOVIE S1, MOV file, 5 MB. Copyright © 2017 Santiago-Tirado et al. 2017 Santiago-Tirado et al. This content is distributed under the terms of the Creative Commons Attribution 4.0 International license.

### Physiological influences on BBB^M^ transit.

**FIG 3:**
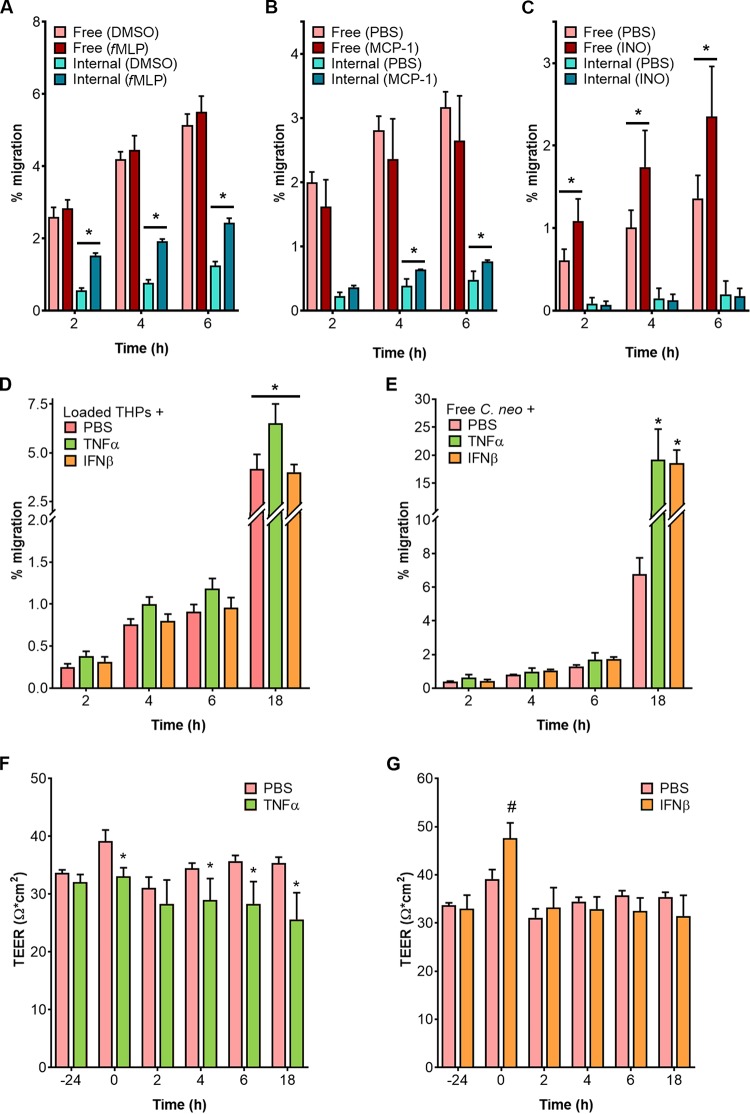
Differential regulation of free and internalized fungal crossing by immune mediators. (A to C) Transmigration of free and internalized fungi in the presence of various chemoattractants, with matched controls. (A) *f*MLP, N-formyl-methionine-leucyl-phenylalanine peptide (100 nM). DMSO, dimethyl sulfoxide. *, *P* < 0.0001 (by Sidak’s multiple-comparison test). (B) MCP-1 (also known as CCL2), monocyte chemoattractant protein-1 (100 ng/ml). *, *P* < 0.03 (by Sidak’s multiple-comparison test). (C) INO, inositol (1 mM). *, *P* < 0.05 (by Sidak’s multiple-comparison test). (D and E) Transmigration assays using BBB pretreated with TNF-α (10 ng/ml) or IFN-β (1 ng/ml) for 24 h before addition of loaded THPs (*, *P* < 0.0003 [for comparisons between TNF-α and other treatments by Tukey’s multiple-comparison test]) (D) or of free fungi (*, *P* < 0.0001 [for comparisons between each compound and PBS by Tukey’s multiple-comparison test]) (E). (F and G) TEER values of model BBBs treated with 10 ng/ml TNF-α (F) or 1 ng/ml IFN-β (G) for 24 h prior to initiation of the study (*t* = 0 h), at which point the medium was replaced with fresh medium without cytokines (*, *P* < 0.01; #, *P* < 0.0002 [all compared to PBS at the same time point by Dunnett’s multiple-comparison test]).

Cryptococcal meningitis outcome correlates with the levels of immune mediators (24–26). MCP-1 (monocyte chemoattractant protein-1) and *f*MLP (N-formyl-methionine-leucyl-phenylalanine) are two chemokines that have been specifically studied for their roles in cryptococcal infection (27–30). MCP-1, found at high levels in the brains of cryptococcal meningitis patients (24–26, 31), recruits monocytes to sites of infection and is a critical factor in the immune response against pulmonary cryptococcosis. Formylated peptides such as *f*MLP are also macrophage activators and are potent chemoattractants for phagocytes; these include neutrophils and dendritic cells, which help control cryptococcal infection (32). Physiological levels of both MCP-1 and *f*MLP significantly stimulated the transit of loaded macrophages, consistent with their chemoattractant function *in vivo*; neither influenced the movement of free fungi (Fig. 3A and B). We next tested inositol, which has been shown to promote the transmigration of free *C. neoformans* across a model BBB (33). The presence of inositol at 1 mM, close to the physiological levels in the brain, increased the transit of free fungi but had no effect on the migration of loaded macrophages (Fig. 3C). Importantly, none of these regulatory molecules impaired BBB^M^ integrity (not shown). Together, these studies showed that Trojan horse and free fungal crossing are independently regulated.

Th1 cytokines, including tumor necrosis factor alpha (TNF-α), are critical for protective immunity and clearance of cryptococcal infection in mice (34). TNF-α and type I interferons (IFNs), such as IFN-β, have been implicated in the control of cryptococcal meningitis in mice and humans (24, 35). These mediators also regulate BBB permeability, especially in response to viral infections (36, 37): TNF-α increases barrier permeability, while IFN-β decreases it. Adding TNF-α or IFN-β to our assays, at levels comparable to those seen in HIV-positive (HIV^+^) patients with cryptococcal meningitis (24–26), yielded small but significant changes in barrier permeability (Fig. 3F and G), consistent with those reports. TNF-α, which increases barrier permeability, also increased the migration of loaded macrophages (Fig. 3D). IFN-β, however, did not affect loaded macrophage migration, possibly because the tightening of the barrier was only transient (Fig. 3G). With free fungi, both TNF-α and IFN-β stimulated transmigration (Fig. 3E), suggesting that these molecules regulate factors beyond barrier permeability that influence free fungal transit.

### Trojan horses enable BBB^M^ transit by fungal mutants that cannot cross alone.

**FIG 4:**
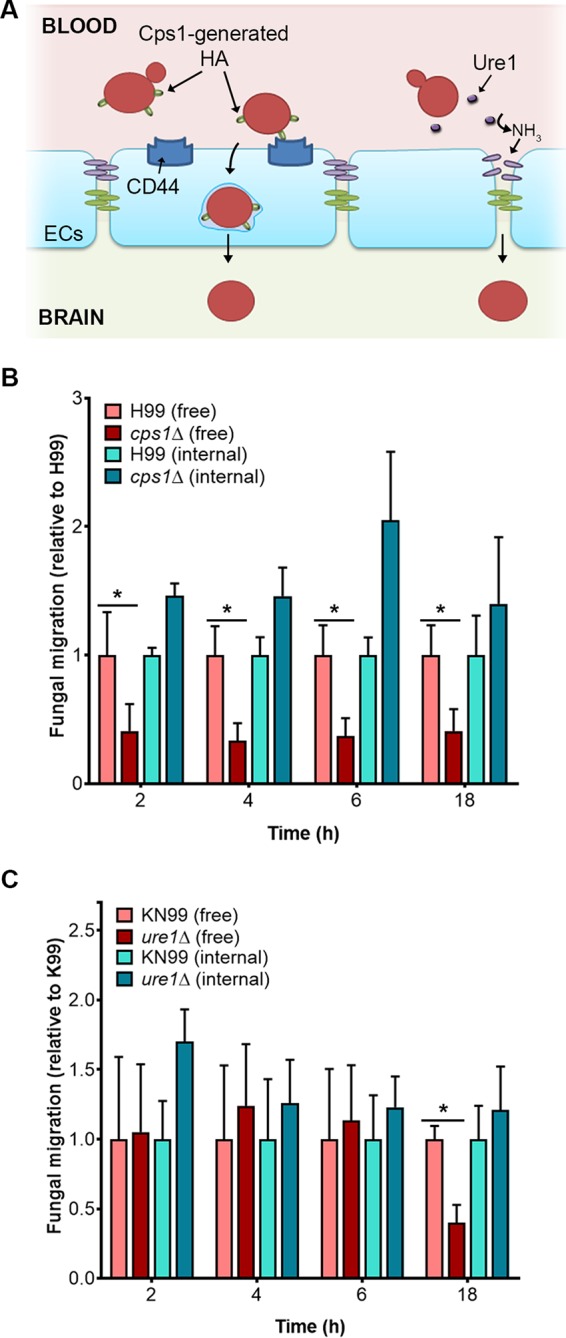
Loaded macrophages provide an alternative route for mutant cryptococci to gain access into the brain. (A) The roles of Cps1 and Ure1 in BBB crossing. HA (green ovals on the surface of the fungi [red]) made by Cps1 is recognized by the endothelial cell (EC) surface receptor CD44 (blue shapes), which triggers endocytosis of the fungal cells. The accumulation of ammonia generated by Ure1 may damage cellular junction proteins, facilitating fungal brain entry. (B and C) Transmigration of free or internalized fungi, comparing wild-type and either *cps1*Δ (B), or *ure1*Δ (C) mutants. Means plus SEM are plotted. * denotes *P* < 0.002 and *P* < 0.0001 in panels B and C, respectively (both determined by Sidak’s multiple-comparison test).

The *CPS1* gene encodes hyaluronic acid (HA) synthase. HA has been implicated in fungal binding to the CD44 surface receptor on hCMECs, which triggers endocytosis and subsequent barrier traversal via a transcellular mechanism (8, 38) (Fig. 4A, left). Consistent with these reports, *cps1*Δ cells had a profound defect in free fungal BBB^M^ crossing (Fig. 4B, red-shaded bars). When these mutants were passengers inside Trojan horse macrophages, however, they traversed the BBB^M^ as efficiently as wild-type fungi (Fig. 4B, blue-shaded bars). The enzyme urease, encoded by *URE1*, is also required for efficient *C. neoformans* brain invasion *in vivo* (11, 12). The mechanism in this case is postulated to be its conversion of urea to ammonia, which could weaken BBB junctions locally and thereby facilitate paracellular crossing (Fig. 4A, right). We indeed observed a defect in free fungal crossing of *ure1*Δ cells (Fig. 4C), although only at late time points, consistent with previous *in vivo* results (39). Notably, TEER values remained stable throughout this experiment (not shown), indicating that the barrier as a whole maintained integrity; it may be that the time-dependent accumulation of ammonia acts only locally or that the monolayer, if disrupted, is perturbed only transiently and recovers rapidly. Again, the mutation did not inhibit the transmigration of internalized fungi (Fig. 4C), confirming that even mutants that are deficient in free fungal entry into the brain can still exploit the Trojan horse route of transit across the BBB. In both studies, some free fungal crossing still occurred with mutant cells, likely due to the multiple possible mechanisms by which free fungi may traverse the BBB (Fig. 4A).

### Trojan horse phagocytes convey cryptococci across transcellular pores.

**FIG 5:**
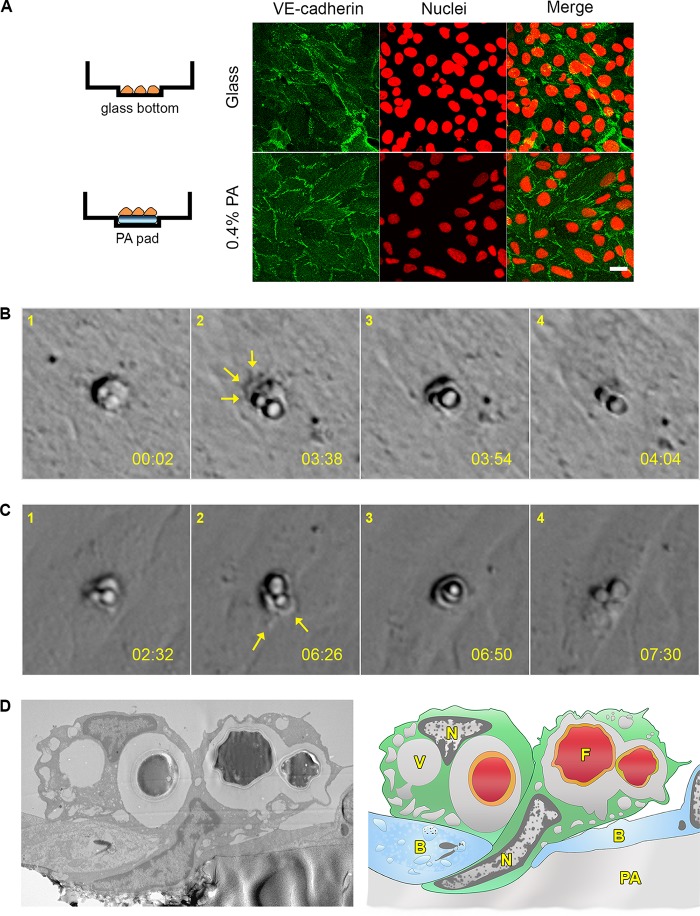
Visualization of Trojan horse crossing by real-time and electron microscopy. (A) hCMEC monolayers were grown on glass or 0.4% PA pads were grown to confluence and fixed, and adherens junctions were stained with anti-VE-cadherin antibody. Nuclei were stained with propidium iodide. (B and C) Two examples of loaded primary human monocytes crossing endothelia, from Movie S2 (B) and Movie S3 (C); see text for details. (D) TEM of a loaded monocyte in the process of transendothelial migration (left), with a corresponding drawing to identify structures (right). N, nucleus; V, vacuole; F, fungal cell; B, brain endothelial cell.

Our confocal microscopy analysis (Fig. 2) provided direct evidence for a Trojan horse mechanism of BBB traversal, in contrast to prior indirect evidence for such transit of a variety of pathogens (40–45). To observe cryptococcal transit over time and to obtain mechanistic insights, we used live-cell microscopy. We first seeded hCMECs on collagen-coated polyacrylamide (PA) hydrogels, which reproduce the physical properties of *in vivo* tissues (46), and monitored the monolayers by phase-contrast microscopy to confirm formation of a single, uninterrupted layer with uniform junctions (Fig. 5A); transmission electron microscopy (TEM) also showed a thin cell monolayer with minimal cytosolic overlap and well-developed junctions (Fig. S2A). To observe intercellular interactions, we then added primary human monocytes loaded with *C. neoformans* (see Materials and Methods) and acquired images every minute for 6 to 16 h. These cells interacted with the endothelial layer more actively than loaded THP-1 cells, with 36% of 60 total events recorded showing Trojan horse transmigration. The typical pattern of Trojan horse crossing that we observed is shown in Fig. 5B and C (images extracted from Movies S2 and S3, respectively). The first image (panel 1) in each sequence shows a loaded monocyte associated with the endothelial cell layer. Host membranes then extend around the loaded phagocyte (panel 2, arrows), a process that follows extensive membrane activity at that site (see Movies S2 and S3) and leads to formation of a transcellular pore (47) (panel 3). After transmigration of the Trojan horse through the pore and pore closure, the fungi can still be seen through the endothelial cell (panel 4), but with a marked loss of refractility compared to the early images. These fungi remained within the original phagocytic cell throughout the process (better visualized in the single-color channels of the movies).

10.1128/mBio.02183-16.3 FIG S2  TEM of hCMECs grown on PA pads. (A) Low-magnification views of PA fibers and the hCMEC monolayer (top row; scale bars = 10 µm) and higher-magnification examples of cellular junctions (bottom row; scale bars = 0.5 µm for the first two images and 2 µm for the last). Arrows indicate the cell junctions. (B) Loaded primary monocytes interacting with hCMECs; scale bars = 2 µm. (C) Free *C. neoformans* within an endothelial cell, highlighting host cell distortion and cytoskeletal elements surrounding the fungus-containing vacuole. Scale bar = 2 µm. Download FIG S2, TIF file, 4.9 MB. Copyright © 2017 Santiago-Tirado et al. 2017 Santiago-Tirado et al. This content is distributed under the terms of the Creative Commons Attribution 4.0 International license.

10.1128/mBio.02183-16.4 MOVIE S2  Live-cell recording of loaded hPBMs interacting with and traversing the hCMEC monolayer. Arrows indicate the start of membrane activity prior to transmigration. Download MOVIE S2, MOV file, 13.2 MB. Copyright © 2017 Santiago-Tirado et al. 2017 Santiago-Tirado et al. This content is distributed under the terms of the Creative Commons Attribution 4.0 International license.

10.1128/mBio.02183-16.5 MOVIE S3  Live-cell recording of loaded hPBM interacting with and traversing the hCMEC monolayer. Arrows indicate the start of membrane activity prior to transmigration. Download MOVIE S3, MOV file, 8.5 MB. Copyright © 2017 Santiago-Tirado et al. 2017 Santiago-Tirado et al. This content is distributed under the terms of the Creative Commons Attribution 4.0 International license.

To observe transmigration events at higher resolution, we prepared samples as described for the video microscopy, incubated the samples for 6 h, washed the samples extensively to remove nonassociated loaded monocytes and media components, and fixed the samples for transmission electron microscopy (TEM). Consistent with the lengthy association between phagocytes and endothelial cells that precedes the rapid progression of transmigration shown in our videos, our TEM images included numerous examples of loaded phagocytes interacting with the endothelial cell monolayer (Fig. S2B) and only occasional instances of transmigration events in progress. One of the latter (Fig. 5D) demonstrates the partial progress of a monocyte, which carries a budding cryptococcal cell, through a transcellular pore. TEM also showed free cryptococcal cells within brain endothelial cells, presumably in the process of transcytosis (Fig. S2C).

We performed similar live-cell studies of loaded THPs and observed various known behaviors of intracellular *C. neoformans*, including intracellular proliferation (not shown), lytic exocytosis (Movie S4, left side), and nonlytic exocytosis (Movie S4, right side) (48). We also observed several instances of cell-to-cell transmission of fungal cells, which has been reported to occur between macrophages (49, 50). In our studies, the fungal movement was from phagocytic to endothelial cells (Movie S5), a novel finding. Free fungi in our movies, probably arising from lytic or nonlytic exit from the loaded macrophages, sometimes associated with or were internalized by endothelial cells, in one case subsequently replicating and then being expelled (Movie S6).

10.1128/mBio.02183-16.6 MOVIE S4  Examples of fungal escape from phagocytes. (Left) A loaded macrophage (arrow) that lyses and allows fungal escape. (Right) Nonlytic escape of fungal cells from a loaded macrophage; arrows indicate the positions of impending fungal extrusion. Download MOVIE S4, MOV file, 11.5 MB. Copyright © 2017 Santiago-Tirado et al. 2017 Santiago-Tirado et al. This content is distributed under the terms of the Creative Commons Attribution 4.0 International license.

10.1128/mBio.02183-16.7 MOVIE S5  Live-cell recording showing cell-to-cell transmission of fungi between a loaded macrophage and an endothelial cell. Yellow arrows indicate the transferred fungi, while magenta arrows indicate extracellular fungal cells as a reference. Download MOVIE S5, MOV file, 6.3 MB. Copyright © 2017 Santiago-Tirado et al. 2017 Santiago-Tirado et al. This content is distributed under the terms of the Creative Commons Attribution 4.0 International license.

10.1128/mBio.02183-16.8 MOVIE S6  Live-cell recording of a free fungal cell moving across the field that is captured and engulfed by an hCMEC. It later buds intracellular, exits nonlytically, and then continues replicating extracellularly. Download MOVIE S6, MOV file, 9.5 MB. Copyright © 2017 Santiago-Tirado et al. 2017 Santiago-Tirado et al. This content is distributed under the terms of the Creative Commons Attribution 4.0 International license.

In 10% of the traversal events that we observed, the fungal cargo appeared to impede monocyte transit across the endothelial cell layer, with the portion of the host cell containing the fungi apparently stuck on the luminal side for the duration of the movie (Movie S7, red arrow). To assess the potential interference with monocyte transit and possible specificity of this process, we compared the transit of empty THPs to that of THPs loaded with either *C. neoformans* or *S. cerevisiae* (Fig. S3A). These studies, whose results we analyzed by both measuring fluorescence (Fig. S3B) and directly imaging and counting cells in the lower chamber (Fig. S3C), suggested that the presence of either yeast slightly inhibited host cell transmigration; there was no difference between the two fungal species in this regard.

10.1128/mBio.02183-16.9 MOVIE S7  Live-cell recording of a loaded macrophage interacting with the hCMEC monolayer, where the presence of fungi apparently prevents complete transmigration during the duration of the movie. Yellow arrows indicate the start of membrane activity prior to transmigration; the red arrow points to the fungal cell that remains above the monolayer; and the white arrow indicates the direction of the futile movement of the host cell, which seems to be stuck. Download MOVIE S7, MOV file, 8.7 MB. Copyright © 2017 Santiago-Tirado et al. 2017 Santiago-Tirado et al. This content is distributed under the terms of the Creative Commons Attribution 4.0 International license.

10.1128/mBio.02183-16.10 FIG S3  Isolation of *S. cerevisiae*-loaded THP-1 cells and the effect of phagocyte loading on transmigration. (A) Flow plots and gating strategy for *S. cerevisiae* loading (essentially the same as that used for *C. neoformans* in the experiment whose results are shown Fig. S1). Gate 1, loaded macrophages with no external fungi; gate 2, phagocytes with adherent CFW^+^ fungi; gate 3, damaged/dead SYTOX^+^ phagocytes. (B) Transmigration of empty THPs or THPs containing either *S. cerevisiae* (*S. cer*) or *C. neoformans* (*C. neo*), assayed by fluorescence. In these studies, model BBBs were generated in 96-well plates with Corning FluoroBlok multiwell inserts, which block light transmission for wavelengths between 400 and 700 nm. This allows DFFDA fluorescence of particles that cross the barrier to be directly measured from below with the FITC filter set of a fluorescence plate reader (Cytation3 system). The data plotted represent the increase in fluorescence compared to control wells without THPs. *, *P* < 0.002 (by Tukey’s multiple-comparison test compared to either loaded population at that time point). (C) Transmigration assays prepared as described for panel B were performed by direct counting. At the desired time points, the inserts were dipped sequentially into HBSS+, fixative (4% formaldehyde–PBS), and HBSS+. A Cytation3 system was then used with the manufacturer’s software to image the bottom of the inserts and count the DFFDA-stained transmigrated THPs. *, *P* < 0.0001 (by Tukey’s multiple-comparison test compared to either loaded population at that time point). Download FIG S3, TIF file, 1.3 MB. Copyright © 2017 Santiago-Tirado et al. 2017 Santiago-Tirado et al. This content is distributed under the terms of the Creative Commons Attribution 4.0 International license.

## DISCUSSION

Because *C. neoformans* thrives intracellularly, the idea of the use of a Trojan horse model for its dissemination to the CNS has been historically appealing and is supported by a variety of indirect studies. However, confirming Trojan horse transit has posed significant experimental challenges, despite its importance for understanding how *C. neoformans* and related species colonize the brain (17, 51, 52). Here we show for the first time that cryptococcal cells maintained within phagocytes cross brain microendothelial barriers, directly demonstrating Trojan horse transit.

Our comparisons of the transit of free fungi to that of phagocytes with internalized yeast showed that free fungi crossed our model BBB more efficiently. However, these *in vitro* studies likely underestimate the role of Trojan horse transit *in vivo* for several reasons. First, free *C. neoformans* does not survive well in serum and is rapidly cleared from the bloodstream following i.v. inoculation in mice (53, 54). Second, unlike free cryptococci, phagocytic cells stick, albeit transiently, to the bottom of the membrane as they cross a model BBB. Although this allowed us to directly demonstrate the transit of loaded macrophages, it likely leads to an underestimation of the internal fungi when the medium in the lower chamber is sampled, as in our assays. In addition, we found that host signals relevant to infection differentially regulate the crossings of free and internalized fungi; these factors may cause greater levels of Trojan horse crossing during infection *in vivo* than we detected *in vitro*. Finally, it has been suggested that free fungi adherent to the luminal side of brain microvasculature may be removed by neutrophils (55), a process that would reduce their transit but would not occur in our system.

The balance of routes used by *C. neoformans* to enter the brain may change during the course of infection. This could be mediated in part by changing levels of host immune regulators, which modulate BBB crossing. These regulators may be influenced by characteristics of the infecting strain of *C. neoformans*, such as the release of the polysaccharide capsule, a major virulence factor. For example, the level of MCP-1, which we found specifically stimulated Trojan horse crossing, is decreased in infections with strains shedding small amounts of capsule compared to strains shedding intermediate levels. Similarly, high levels of TNF-α, which stimulated both Trojan horse and free fungus traversal, correlate with infecting strains that have high levels of capsule shedding (4). The balance of routes could also be influenced by the progression of the disease itself. In our work, the greatest specific effect on Trojan horse traversal was caused by *f*MLP. Formyl peptides such as *f*MLP stimulate not only leukocyte chemotaxis but also degranulation, superoxide production, and activation of integrins for cell adhesion, all of which promote inflammation. Both *in vivo* and *in vitro* studies have suggested that endothelial damage occurs late in cryptococcal infection (13, 54). Such damage would result in release of formyl peptides, again potentially shifting the balance of CNS entry routes.

**FIG 6:**
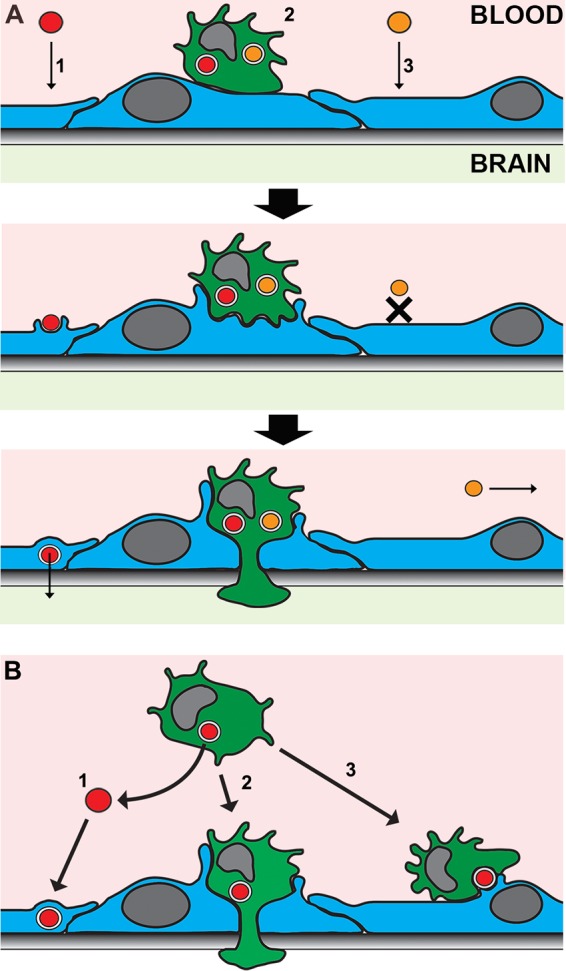
Mechanisms of brain infection by *C. neoformans*. (A) Depicted are wild-type fungi (red ovals) crossing the BBB either free (1) or within Trojan horse phagocytes (2). Mutant fungi (orange ovals) cannot cross alone (3) but can use Trojan horse transit as an alternative route. See text for details on transendothelial pore formation for Trojan horse transit. (B) Loaded phagocytes potentially contribute to brain invasion by pathways that do not involve true Trojan horse transit (2). Phagocytes can bring the fungus to the CNS internally and then exit the phagocyte by nonlytic exocytosis and cross the BBB as free yeast by transcytosis (1). Finally, we observed two instances of direct cell-to-cell transfer of fungal cells from phagocytes to endothelial cells (3), supporting the hypothesis of a “taxi” mechanism, where loaded phagocytes deliver fungal cells directly into brain endothelial cells. Pink, blood vessel lumen; pale green, brain parenchyma; blue cells, brain endothelial cells; green cells, infected phagocytes; shaded gray rectangle, the extracellular matrix that forms the BBB basal membrane.

Our live-cell microscopy of Trojan horse crossing showed a distinctive sequence of events (Fig. 5). These events are consistent with the process of transendothelial pore formation, cartooned in Fig. 6, where the phagocyte (green) essentially crosses through a “donut hole” that forms in the endothelial cell, without ever entering its cytosol. This mechanism has not been considered in the cryptococcal literature, although it is consistent with models for inflammatory cell crossing in the brain. Due to the presence of extremely tight junctions in brain endothelia, transendothelial pore transit accounts for up to 90% of cellular crossing of the BBB, in contrast to other endothelia, where most crossing occurs paracellularly (56). A minor role for paracellular transit is consistent with the stable TEER values measured during our assays. It is also consistent with our observations revealing that, in several videos where cell boundaries are clear, crossing occurred at the cell body rather than at the edges (Movies S2 and S3 and data not shown). While it is conceivable that a particle could cross the endothelial barrier without perturbing TEER (if the apical junction sealed before the basal one was breached), we consider this unlikely because of the kinetics of crossing and the large size of the fungus relative to the thickness of the endothelial layer (Fig. S2C).

We also recorded other behaviors that could contribute to *C. neoformans* BBB crossing. These included nonlytic escape from phagocytes and a novel process of fungal movement from phagocytes directly into endothelial cells; both of these suggest additional modes of BBB traversal where phagocytes participate without acting as Trojan horses. For example, phagocytes could bring cryptococci to the BBB, where they would either exit and cross transcellularly on their own or be delivered directly into the endothelia by cell-to-cell transfer (Fig. 6B and Movies S4 and S5). Although Trojan horse crossing was more frequent (36% of recorded events) than nonlytic exocytosis or direct transfer (26% or 3%, respectively) in our system, the latter mechanisms may contribute to brain entry *in vivo* (19, 57).

The presence of internalized fungi, whether *C. neoformans* or model yeast, had a modest inhibitory effect on phagocyte traversal (Fig. S3). Our movies suggest that this may be because they contain relatively large and rigid structures that are not easily accommodated by the endothelial pore; this idea is also supported by a correlation between this inhibition and the number of internalized fungi per cell (not shown). The effect of the physical parameters of the yeast is also evident in our TEM observations of free fungal transit. The thin brain endothelium is dramatically distorted by fungal entry, such that the yeast contacts both cell surfaces (Fig. S2C). The long fibers surrounding the internalized cryptococci are presumably cytoskeletal elements, which could potentially trigger expulsive events toward either side, such as those shown in Movie S6, where the fungal cell is internalized by and subsequently exits from the same face of the endothelial layer.

We found that fungal cells that cannot cross the BBB on their own (Fig. 6, orange ovals) can still reach the brain inside phagocytes, at levels comparable to those seen with normal fungi. This is likely to be relevant to pathogenesis, as exemplified by mutants lacking Cps1, which produces a ligand for endothelial cell CD44 (Fig. 4A). Although independent transmigration of these mutant fungi is reduced ~50% compared to that of wild-type fungi *in vitro*, wild-type infections of CD44^−/−^ mice show a smaller reduction in brain burden and a minimal effect on virulence (58). This can be explained by a model where most circulating fungal cells are inside phagocytes. These fungi would not need the CD44 interaction to efficiently cross the BBB, consistent with the *in vivo* observations and supporting the idea of a role for Trojan horse crossing *in vivo*. These findings highlight the need to consider internalized fungi in assessments of antifungal agents and the potential value of identifying compounds that inhibit intracellular replication of cryptococci, as in a recent screen (59).

Any intracellular pathogen that can survive within phagocytes can potentially exploit them as Trojan horses for dissemination or delivery into target organs, although some may use other mechanisms, as suggested recently for *Toxoplasma gondii* (60). Our work shows the importance of dissecting these mechanisms for *C. neoformans* and other neuropathogens and provides methods and approaches for doing so.

## MATERIALS AND METHODS

### Mammalian cells and growth conditions.

The human monocytic cell line THP-1 (TIB-202, from the ATCC) was cultured and differentiated as described in reference 61. These cells were negative for mycoplasma. For the model BBB, we obtained the immortalized human brain endothelial cell line hCMEC/D3, described and reviewed in reference 22. These cells were obtained from Babette Weksler (Cornell University Medical College) and were grown as described in reference 23. Primary blood monocytes were isolated by negative immunoselection (Pan Monocyte isolation kit; Miltenyi Biotec, Inc.) from peripheral blood mononuclear cells (PBMCs) that were obtained from leukoreduction (LRS) chambers following routine platelet donations at the apheresis center at Washington University School of Medicine. The cells were kept in suspension by growth in ultra-low-adherence flasks (Corning) in human peripheral blood monocyte (hPBM) medium (RPMI medium with l-glutamine and sodium bicarbonate, 10% fetal bovine serum [FBS], 1 mM sodium pyruvate, 1× penicillin-streptomycin [PenStrep], 1% nonessential amino acids [NEAA], and 10 mM HEPES [pH 7]).

### Fungal strains.

Most studies used *C. neoformans* strain KN99α (62), provided by Joe Heitman, or mCherry-expressing KN99α from Jennifer Lodge and Raj Upadhya. A *ure1*Δ strain in the KN99α background was from the 2015 Madhani partial-deletion collection (Fungal Genetics Stock Center, University of Missouri—Kansas City, Kansas City, MO). A *cps1*Δ strain in the H99α background was obtained from an earlier Madhani collection (63); H99α was used as a control for experiments performed with this mutant. *S. cerevisiae* strain BY4741 (64), provided by Michael Brent, was transformed with an integrative plasmid expressing cytoplasmic mCherry (from Addgene).

### Fungal uptake by phagocytes.

THPs seeded at 1.67 × 10^5^ cells/ml and grown in T175 flasks were labeled with 1 µM DFFDA succinimidyl ester (DFFDA-SE) (CellTrace Oregon Green; Thermo Fisher Scientific) and challenged with mCherry-expressing fungi or fungi stained with 15 µM DDAO-SE (CellTrace Far Red; Thermo Fisher Scientific) at a multiplicity of infection (MOI) of 3. A 2.5-h incubation at this MOI yielded close to 1 yeast per host cell (Fig. S1A), with more than half of the THPs infected and relatively few externally adherent fungi (Fig. S1B). For some uptake studies, fungi were opsonized (30 min at 37°C with rotation) with 40% fresh serum (from healthy individuals at the Washington University School of Medicine; Institutional Review Board [IRB] 201101914) before being exposed to phagocytes. After 2.5 h, flasks were washed twice with 2 mM EDTA–phosphate-buffered saline (PBS) to remove free and loosely adherent fungi and the THPs were detached with Cellstripper (Corning), a nonenzymatic cell dissociation solution. The cells were then washed once with medium lacking phenol red and resuspended in Presort buffer (BD Biosciences) with 10 µg/ml calcofluor white (CFW) to stain external fungi and 5 nM SYTOX red (using mCherry-expressing fungi) or SYTOX orange (using DDAO-stained fungi) as a host cell viability dye. A similar approach was used with primary blood monocytes (not shown), except that initial washes were done by centrifugation and uptake was performed in medium without phenol red at an MOI of 1 for at least 3 h; infected cells were then sedimented and resuspended in Presort buffer containing CFW and SYTOX red as described above.

### Flow cytometry.

To optimize phagocytosis assays, we analyzed uptake reactions on an LSRII instrument (BD Biosciences). Cell sorting was performed on a FACSAria II system (BD Biosciences) following standard procedures with appropriate precautions for a biosafety level 2-plus (BSL-2^+^) organism. The gating strategy is shown in Fig. S1C to F (and the last step only is shown in Fig. 1A); at least 10^6^ cells were routinely collected from gate 1 in Fig. 1A and Fig. S1F. All results were analyzed and formatted for presentation using FlowJo software (Treestar).

### *In vitro* BBB.

BBB^M^ (Fig. 1C) examples were generated as described in reference 23 with some modifications. Briefly, hCMEC/D3 were seeded at 5 × 10^4^ cells/cm^2^ (for 0.9-cm^2^ permeable inserts) or 6 × 10^4^ cells/cm^2^ (for 0.33-cm^2^ inserts) on the apical side of 8-µm-porosity inserts (BD Falcon, Corning) that had been coated with 150 µg/ml Cultrex rat collagen I (R&D Systems) for at least 1 h at 37°C prior to washing with PBS and adding the cells. The cells were grown for 4 to 5 days in EBM2 (Lonza) supplemented with FBS (5%), penicillin-streptomycin (1%), hydrocortisone (1.4 μM), ascorbic acid (5 μg/ml), chemically defined lipid concentrate (1%), HEPES (10 mM), basic fibroblast growth factor (bFGF) (1 ng/ml), and human vascular endothelial growth factor (hVEGF) (2 ng/ml). At 24 h prior to the assays (when the monolayer was already confluent), the medium was replaced with differentiation medium (as described above but with 1% FBS and no growth factors). TEER was measured with an EVOM2 (WPI) with either an STX2 electrode (for 12-well plates) or an STX100 electrode (for 24-well plates). We routinely achieved values of ~80 Ω ⋅ cm^2^ for 0.9-cm^2^ inserts and ~60 Ω ⋅ cm^2^ for 0.33-cm^2^ inserts with 8-µm-pore-size membranes. We observed much lower TEER values when we used inserts with 0.4-µm pores (not shown). These results were similar to what was found in a recent study, although even those barriers were reported to still maintain integrity with respect to large molecules (65).

### Transendothelial migration assays.

For studies with free fungi, log-phase *C. neoformans* fungi were washed twice in PBS and 10^8^ cells were resuspended and incubated in 40% fresh human serum–PBS for 30 min at 37°C. The cells were collected by centrifugation and resuspended at 2 × 10^6^ cells/ml in migration media (RPMI medium containing 1% FBS), and 500 μl of the suspension was added to the top of a permeable insert (usually 0.9-cm^2^ permeable inserts in 12-well plates), which was then moved into a new plate with 1.5 ml of migration medium/well. At each time point, TEER was measured. Inserts were then moved to new plates with prewarmed migration medium, and aliquots of the prior lower chamber were spotted on yeast extract-peptone-dextrose (YPD) plates for CFU quantification. For studies involving loaded macrophages, 10^5^ particles (free fungi, loaded macrophages, or a 1:1 mix of free fungi and empty macrophages)–300 µl of migration media was added to the top of a 0.33-cm^2^ Corning HTS insert (in a fixed-format 24-well plate to allow plate centrifugation without cross-contamination). Prior to each sampling, the plate was subjected to centrifugation (1,000 rpm, 5 min, 37°C) and the insert assembly was moved to a new plate containing 1 ml migration medium/well; aliquots from the prior wells were spotted on YPD plates for CFU quantification. In some studies, chemoattractants or cytokines were present in the bottom chamber of each well at the concentrations noted in the text. Excluding Fig. 1, all experiments incorporated 4 technical replicates for each condition tested. This allowed experiments to be performed in individual multiwell plates, eliminating plate-to-plate variation for this complex system. Each experiment was performed at least twice independently, with similar results; one example is shown. Details of replicates prepared as described for Fig. 1 are provided in the corresponding legend.

### Direct visualization of insert membranes.

BBB transit assays were performed as described above, the inserts were removed from 12-well plates and immersed twice in HBSS+ (Hanks balanced salt solution containing 1% glutamine, 1% PenStrep, 140 mg/liter CaCl_2_, 100 mg/liter MgCl_2_, 100 mg/liter MgSO_4_, and without phenol red), after which both sides were fixed with 4% formaldehyde–PBS (10 min, room temperature [RT]). The fixed inserts were washed in HBBS+ twice and both sides stained with CellMask Deep Red Plasma membrane stain (Thermo Fisher Scientific) (1:10,000 in PBS; 10 min, RT) and washed twice in HBBS+. The inserts were then excised from the plastic housing using 10-mm-diameter skin biopsy specimen punches and placed monolayer side up on a bubble of mounting media on a microscope slide. Another few microliters of mounting media and a coverslip were then applied. After the mounting media had cured, the membrane was viewed on a Zeiss LSM 510 confocal laser scanning system. To image cells directly, we used Fluoroblok plates and media without phenol red. Wells were washed once with HBSS+ and fixed as described above, and the lower part of the insert was imaged without removal from the plate using a Cytation3 cell imaging multimode plate reader (Bio-Tek Instruments, Inc.).

### Soft substrate preparation and microscopy.

PA pads were prepared in 35-mm-diameter glass-bottomed dishes as previously described (66), coated with 50 µg/ml rat collagen, and used as the substrates for hCMEC monolayers that reproduce the physical properties of *in vivo* tissues (46). For staining of junctions, monolayers were washed with HBSS+ and fixed either with 100% cold methanol (for staining with anti-vascular endothelial cadherin [anti-VE-cadherin] [clone 55-7H1; BD Biosciences]) or with 4% formaldehyde–HBSS+ (for staining with ZO-1 [clone 1A12; Invitrogen] or with claudin-5 [clone 4C3C2; Invitrogen]; not shown). The dishes were then either incubated for 6 h, washed, and processed for TEM or placed into an environmental chamber (Stage Top incubator; Tokai Hit, Shizuokaken, Japan) at 37°C and 5% CO_2_ on an inverted microscope (Olympus IX72) for video recording, with images (DIC [differential inference contrast], fluorescein isothiocyanate [FITC], and Texas Red channels) captured every minute for 6 to 16 h.
