# Supplementary figures and images for: Trojan Horse Transit Contributes to Blood-Brain Barrier Crossing of a Eukaryotic Pathogen
Source: mBio. 2017 Jan 31;8(1):e02183-16. doi: 10.1128/mBio.02183-16 (PMC5285505; doi:10.1128/mBio.02183-16)

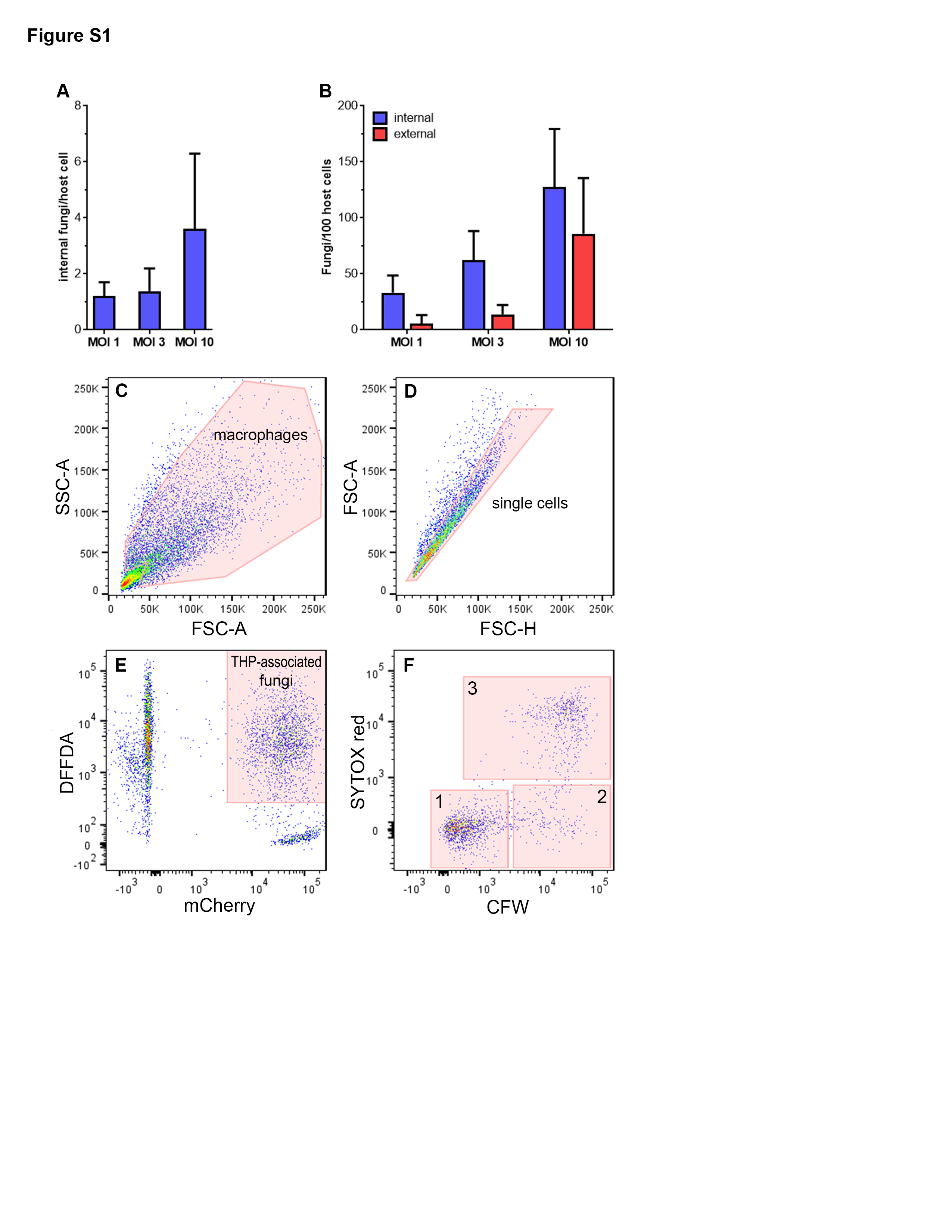

Supplement: FIG S1 [file mbo001173168sf1.tif]

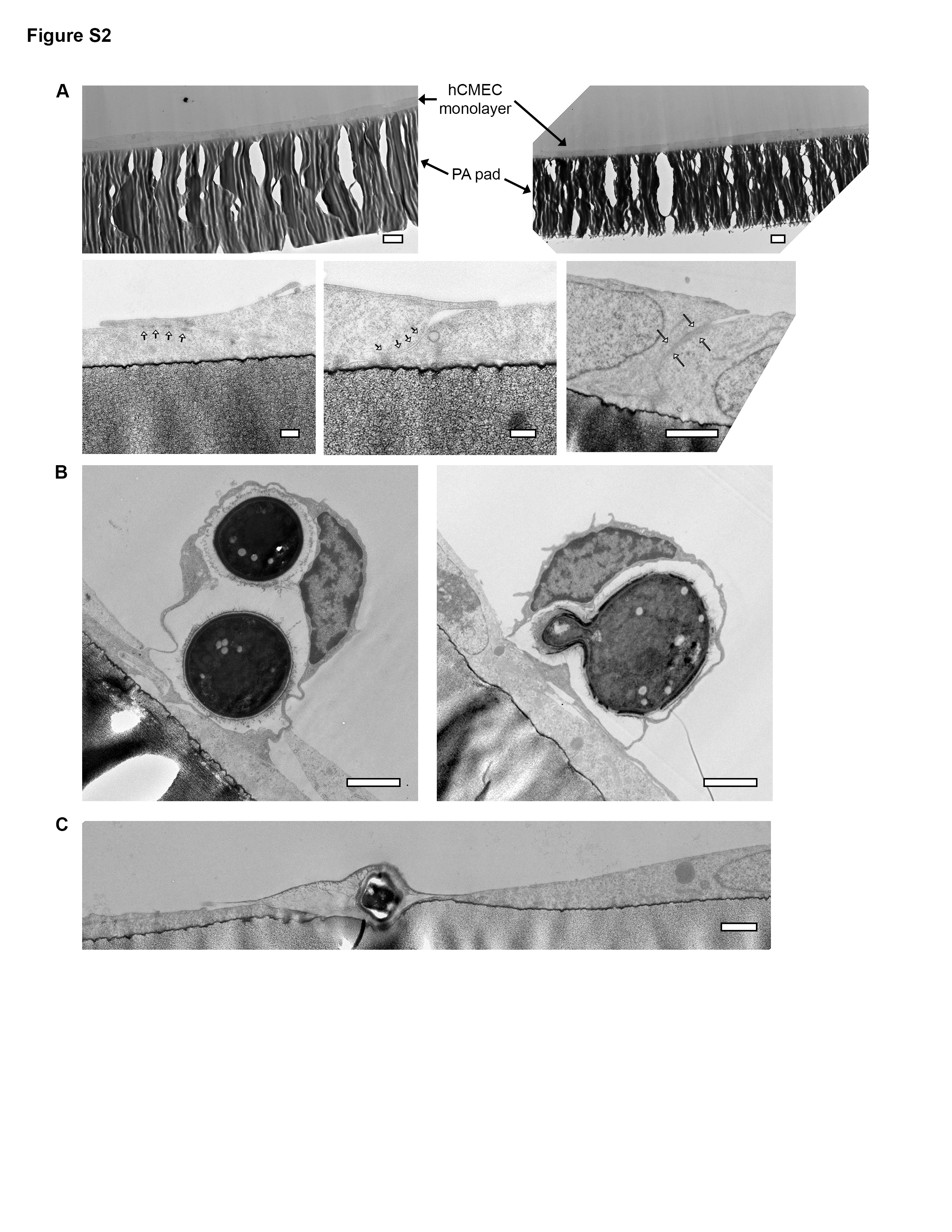

Supplement: FIG S2 [file mbo001173168sf2.tif]

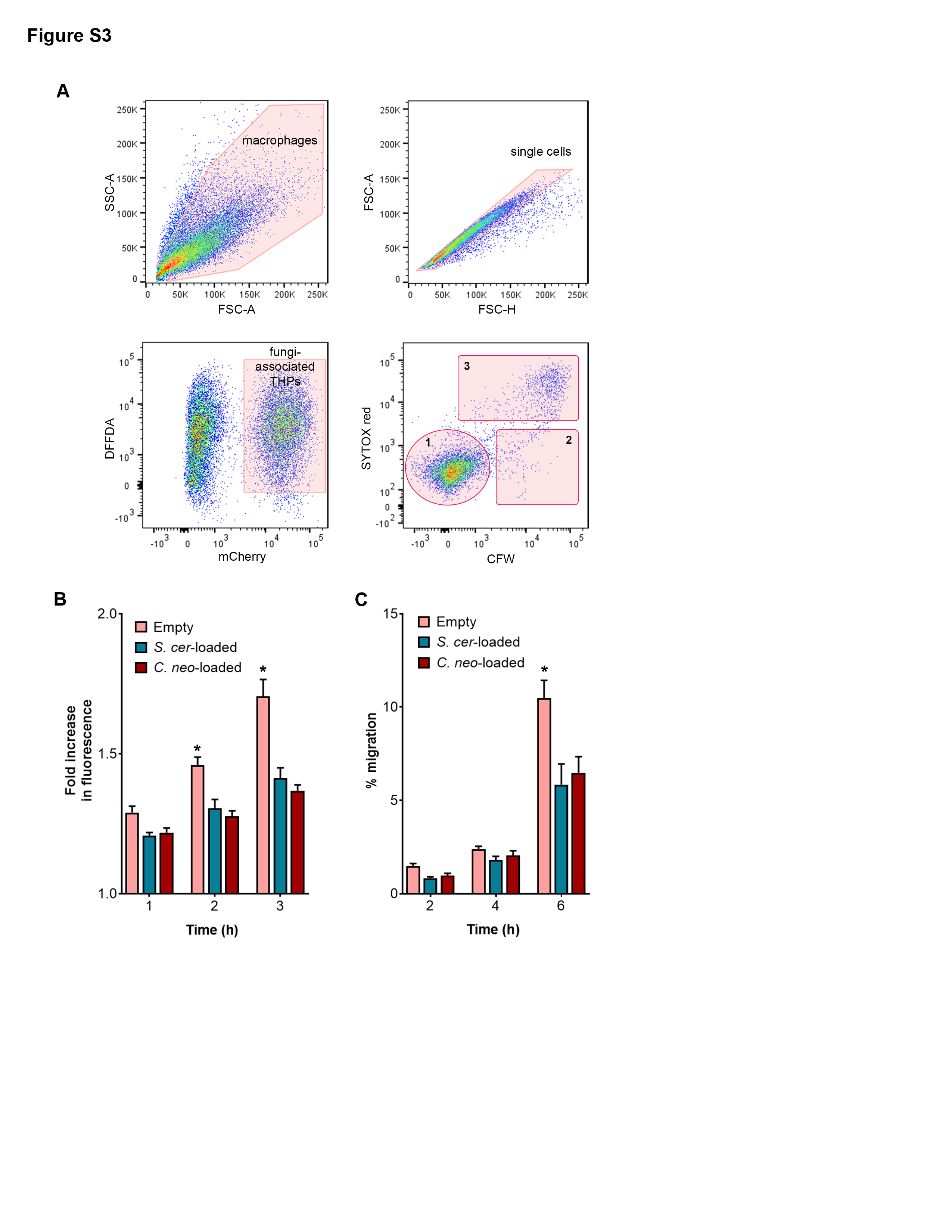

Supplement: FIG S3 [file mbo001173168sf3.tif]
